# Optimizing PH Domain-Based Biosensors for Improved Plasma Membrane PIP_3_ Measurements in Mammalian Cells

**DOI:** 10.3390/cells14141125

**Published:** 2025-07-21

**Authors:** Amir Damouni, Dániel J. Tóth, Aletta Schönek, Alexander Kasbary, Adél P. Boros, Péter Várnai

**Affiliations:** 1 Department of Physiology, Faculty of Medicine, Semmelweis University, 1094 Budapest, Hungary; amir.damouni@stud.semmelweis.hu (A.D.); toth.daniel@semmelweis.hu (D.J.T.);; 2 HUN-REN-SU Molecular Physiology Research Group, Hungarian Research Network and Semmelweis University, 1094 Budapest, Hungary

**Keywords:** phosphoinositide (PI), phosphatidylinositol 3,4,5-trisphosphate (PIP_3_), pleckstrin homology (PH) domain, biosensor, general receptor for phosphoinositides 1 (GRP1), ADP ribosylation factor 6 (Arf6), protein kinase B (Akt), bioluminescence resonance energy transfer (BRET), confocal microscopy

## Abstract

Phosphoinositide-binding pleckstrin homology (PH) domains interact with both phospholipids and proteins, often complicating their use as specific lipid biosensors. In this study, we introduced specific mutations into the phosphatidylinositol 3,4,5-trisphosphate (PIP_3_)-specific PH domains of protein kinase B (Akt) and general receptor for phosphoinositides 1 (GRP1) that disrupt protein-mediated interactions while preserving lipid binding, in order to enhance biosensor specificity for PIP_3_, and evaluated their impact on plasma membrane (PM) localization and lipid-tracking ability. Using bioluminescence resonance energy transfer (BRET) and confocal microscopy, we assessed the localization of PH domains in HEK293A cells under different conditions. While Akt-PH mutants showed minimal deviations from the wild type, GRP1-PH mutants exhibited significantly reduced PM localization both at baseline and after stimulation with epidermal growth factor (EGF), insulin, or vanadate. We further developed tandem mutant GRP1-PH domain constructs to enhance PM PIP_3_ avidity. Additionally, our investigation into the influence of ADP ribosylation factor 6 (Arf6) activity on GRP1-PH-based biosensors revealed that while the wild-type sensors were Arf6- dependent, the mutants operated independently of Arf6 activity level. These optimized GRP1-PH constructs provide a refined biosensor system for accurate and selective detection of dynamic PIP_3_ signaling, expanding the toolkit for dissecting phosphoinositide-mediated pathways.

## 1. Introduction

Phosphoinositides (PIs) are essential structural and functional components of biological membranes. These amphipathic molecules are composed of a glycerol backbone attached to two non-polar fatty acid tails, which vary in length, and a polar inositol head group linked via a phosphate group. Enzymatic modifications of the inositol ring result in seven phosphatidylinositol phosphates (PIPs), each characterized by distinct phosphorylation patterns. These PIPs are dynamically interconverted by the actions of specific kinases and phosphatases [1,2]. Present ubiquitously in all human cell biomembranes, phosphoinositides are integral not only to membrane architecture but also to functions such as membrane identification and signaling, orchestrating a vast array of cellular activities [3]. Many of their roles are facilitated through interactions with proteins via specific lipid-binding domains, such as pleckstrin homology (PH) domains [4,5].

PH domains are approximately 120-amino-acid long regions commonly found in a wide range of intracellular signaling proteins [6]. These domains differ significantly in their PIP binding affinity and specificity, with certain PH domains binding to only one and others capable of binding to multiple PIP species. Notably, only a small fraction (approximately 10%) of the 234 PH domains in the human proteome bind strongly to phosphoinositides [7]. Their selective binding capacity enables PH domains to direct proteins to defined membrane compartments, thereby contributing to the spatial and temporal regulation of signaling pathways. PH domains are found in a diverse array of signaling molecules, including kinases, guanine nucleotide exchange factors (GEFs), GTPase-activating proteins (GAPs), phospholipases, and other proteins, underscoring their broad functional spectrum in cellular dynamics [8]. Interestingly, although many PH domain-containing proteins possess multiple PH domains, typically only one is actively involved in PIP binding [9]. PH domains with high specificity and affinity for particular PIPs form the basis of lipid biosensors, which are widely employed to monitor dynamic changes in phosphoinositide levels and to investigate lipid-mediated signaling processes [10].

PH domains are well known to mediate plasma membrane (PM) localization primarily through their interactions with phosphoinositides such as phosphatidylinositol 3,4,5-trisphosphate (PIP_3_). However, it is also well established that, for several PH domains, lipid binding alone is not sufficient to account for their membrane targeting. In these cases, additional lipid-independent interactions with membrane-associated proteins or structural elements contribute significantly to their localization, a concept referred to as coincidence detection [11,12]. For example, in the case of dynamin, the affinity of its PH domain for PIs is insufficient to direct proper membrane localization by itself, yet its distribution remains regulated by inositol lipid signaling, suggesting the involvement of additional interactions [13,14,15]. Furthermore, overexpression of isolated PH domains can competitively displace endogenous membrane-associated proteins, thereby inhibiting their function. Interestingly, mutations in PH domains that preserve lipid binding but disrupt lipid-independent interactions, reduce this dominant negative effect. These findings support a model in which both lipid binding and lipid-independent interactions are essential for accurate PH domain localization and functional regulation at the membrane, as was demonstrated in the cases of protein kinase B (PKB or Akt) and general receptor for phosphoinositides-1 (GRP1) [16].

GRP1 is a GEF of the cytohesin family that activates ADP-ribosylation factor (Arf) monomeric GTPases and plays a critical role in vesicular trafficking and cytoskeletal remodeling in mammalian cells [17]. GRP1 contains a PH domain that specifically binds PIP_3_, a prerequisite for its localization to the plasma membrane and proper cellular function [18]. While negatively charged phospholipids such as phosphatidylserine may contribute to its membrane localization by generating a weak, transient association, they are insufficient for stable binding on their own [19,20,21]. The isolated GRP1-PH domain has been widely employed as a biosensor for detecting plasma membrane-associated PIP_3_ [10,22,23]. However, its membrane association has been shown to depend not only on PIP_3_ binding but also on protein–protein interactions. This was demonstrated by two point mutations at a putative interaction site outside of the lipid-binding pocket, I307E and K340L, which preserve lipid binding yet abolish the ability of the PH domain to exert dominant negative effects [16]. The required non-lipid interaction partner was later identified as the active, GTP-bound form of Arf6 [24].

Akt is a serine/threonine kinase central to the phosphoinositide 3-kinase (PI3K) signaling pathway, regulating key cellular processes such as proliferation, apoptosis, transcription, and glucose metabolism [25,26]. Its activation is triggered by receptor tyrosine kinases (RTKs), which activate PI3K and lead to the production of PIP_3_ at the PM [27]. Akt translocates to the PM via its PH domain by binding PIP_3_, where it becomes activated and subsequently phosphorylates numerous target proteins in the cytosol, nucleus, and at the membrane [28]. In addition to PIP_3_, the PH domain of Akt, specifically that of Akt2, can also bind PI(3,4)P_2_ [29], and Akt has also been shown to interact with phosphatidylserine at a distinct site that enhances PIP_3_ binding [30]. Moreover, Akt is also thought to be involved in protein–protein interactions independent of lipid binding, although the interacting partners remain unidentified. Mutations at threonine 34 in the PH domain, such as T34L and T34F, disrupt this non-lipid interaction without affecting lipid binding, as indicated by a lack of dominant negative effects [16].

In this study, we leveraged specific mutations in the PIP_3_-specific PH domains of Akt and GRP1 that selectively disrupt protein-mediated interactions while preserving lipid binding, with the aim of improving biosensor specificity for PIP_3_. Using bioluminescence resonance energy transfer (BRET), we evaluated the plasma membrane localization of these biosensors in HEK293A cells under various stimulation conditions. While Akt-PH mutants displayed minimal deviation from wild-type behavior, GRP1-PH mutants exhibited markedly reduced membrane association both at baseline and following stimulation with epidermal growth factor (EGF), insulin, or vanadate. To enhance PIP_3_ avidity and biosensor performance, we further developed tandem mutant GRP1-PH constructs, enabling robust detection of PIP_3_ dynamics. In addition to BRET-based assays, we demonstrated the utility of these optimized biosensors in confocal microscopy. Finally, we examined the influence of Arf6 activity and found that, unlike their wild-type counterparts, the mutant GRP1-PH sensors localized to the membrane independently of Arf6, further improving their specificity for PIP_3_ detection.

## 2. Materials and Methods

### 2.1. Materials

All molecular biology materials, including Lipofectamine 2000, were purchased from Thermo Fisher Scientific. Coelenterazine-H was supplied by Regis Technologies (Morton Grove, IL, USA), and insulin was obtained from Eli Lilly (Indianapolis, IN, USA). Unless noted otherwise, additional chemicals and reagents were sourced from Sigma–Aldrich (St. Louis, MO, USA).

### 2.2. DNA Constucts

The PH domain constructs utilized in this work included the following amino acid regions: human GRP1 (residues 267–399; AJ005197), human Akt1 (residues 1–164; NM_005163), and human Btk (residues 1–177; BC109079) [31,32]. To generate GRP1-PH biosensors harboring the I307E and K340L point mutations, the original PH domain sequences described in our earlier study [16] were substituted with a wild-type version using BglII and EcoRI restriction enzymes [33]. For constructing the tandem GRP1-PH biosensor (L10-mVenus-T2A-NES-Luciferase-GRP1-2xPH), a second PH domain sequence was inserted between the first PH and the luciferase coding region at the BglII site, with the two domains linked via an SSREGS peptide. Similarly, a tandem Btk-PH biosensor (L10-mVenus-T2A-Btk-2xPH-Luciferase) was created by introducing a second PH domain sequence between the initial PH and luciferase regions at the AgeI site, joined by a DPPVAAGAGGAG linker [34]. For fluorescent labeling, both single and tandem GRP1-PH constructs were inserted between the BglII and EcoRI sites of the pEGFP-C1 vector, in which GFP was replaced by an N-terminal NES (ALQKKLEELELDEA)-tagged Venus fluorescent protein.

The human EGF receptor, non-internalizing rat type-1 angiotensin receptor (AT1R Δ319) and sensors for phosphatidylinositol 4-phosphate (PI4P) (L10-Venus-T2A-Luc-SidM-2xP4M) and phosphatidylinositol 4,5-bisphosphate [PI(4,5)P2] (L10-Venus-T2A-PLCd1-PH-Luc) were described [16,35,36]. Arf6 constructs were a kind gift from Julie Donaldson, NHLBI, NIH (Bethesda, MD, USA) [24].

### 2.3. Cell Culture

All experiments were conducted using HEK293A cells, obtained from Thermo Fisher Scientific (Waltham, MA, USA) (Cat. No. R70507). The cells were cultured in DMEM (Gibco (Waltham, MA, USA) 11995065) containing 10% fetal bovine serum (Biosera (Cholet, France)), FB-1200), along with 50 units/mL penicillin and 50 µg/mL streptomycin (Gibco (Waltham, MA, USA), 15140-122). Cultivation was carried out in 10 cm plastic culture dishes at 37 °C in a humidified incubator with 5% CO_2_.

### 2.4. BRET Measurements

HEK293A cells were detached using 0.25% trypsin (Lonza (Basel, Switzerland), Cat. No. BE17-160E) and seeded into white 96-well plates (Greiner Bio-One (Kremsmünster, Austria), Cat. No. 655083) pre-coated with 0.001% poly-L-lysine for 1 h. Cells were plated at a density of 6 × 10^4^ per well. Co-transfection was performed using Lipofectamine 2000 (0.25 μL per well) and 0.03–0.04 μg of total plasmid DNA per well, diluted in Opti-MEM reduced serum medium (Gibco (Waltham, MA, USA), Cat. No. 31985047). To overcome the variability associated with endogenous EGF receptor (EGFR) expression in HEK293-derived cell lines, in addition to the BRET sensors, cells were also transfected with EGFR. After 28 h, BRET measurements were performed following a 5 h serum starvation period. Before measurements, the medium was replaced with 50 μL of modified Krebs–Ringer buffer (120 mM NaCl, 4.7 mM KCl, 1.2 mM CaCl_2_, 0.7 mM MgSO_4_, 10 mM glucose, and 10 mM Na-HEPES, pH 7.4) and incubated at 37 °C for 1 h. Measurements were conducted at 37 °C using a Thermo Fisher Scientific (Waltham, MA, USA) Varioskan LUX Multimode Microplate Reader. To begin the measurements, 40 μL of coelenterazine-H (a membrane-permeable luciferase substrate) was added to each well in a final concentration of 5 μM. Luminescence signals were then captured using 480 nm and 530 nm emission filters, with an acquisition time of 250 ms per wavelength. Additional compounds were introduced in 20 μL volume dissolved in modified Krebs–Ringer buffer. For experiments involving vanadate, a fresh solution was prepared before each run by combining 30 μM hydrogen peroxide with 100 μM sodium orthovanadate in a 1:1 ratio. All measurements were conducted in duplicate biological replicates. BRET ratios, used to monitor changes in plasma membrane lipid content, were determined by dividing the emission intensity at 530 nm by that at 480 nm. Since absolute ratio values depended on sensor expression levels, in some experiments these ratios were normalized with resting levels set to 100%, while 0% was determined from experiments using a cytoplasmic luciferase construct alone.

### 2.5. Confocal Microscopy

HEK293A cells were seeded at a density of 3 × 10^4^ cells per well onto IBIDI 8-well μ-Slides (Cat. No. 80826) pretreated with 0.001% poly-L-lysine for 1 h. Cells were cultured in DMEM at 37 °C for 24 h before transfection. For transfection, the medium was replaced with 200 μL Opti-MEM containing the indicated DNA constructs (0.2 μg total DNA/well) and 0.33 μL/well Lipofectamine 2000. After 6 h, the transfection medium was replaced with 300 μL supplemented DMEM. Serum-starved cells (5 h) were imaged 24–26 h post-transfection using a Zeiss LSM 710 laser confocal microscope equipped with a 63×/1.4 oil-immersion objective, heated to 30 °C. Prior to imaging, medium was changed to 200 µL/well modified Krebs-Ringer buffer (described above) at room temperature. For stimulation, EGF was added in an additional volume of 100 μL. Image analysis was conducted using Fiji 1.54p and Photoshop C3 (Adobe), with only linear adjustments applied during processing. After background subtraction, fluorescent pixel intensity changes in various compartments of the cells were measured by Fiji.

### 2.6. Mathematical and Statistical Analysis

Data analysis was carried out using SigmaPlot version 10.0. The particular statistical methods employed are described in the corresponding figure legends. Quantitative results are expressed as mean ± standard deviation, based on a minimum of three separate experiments.

## 3. Results

### 3.1. The PM PIP_3_ Sensing Ability of the GRP1-PH- and Akt-PH-Based Biosensor Mutants Compared to the Wild Type

**Figure 1:**
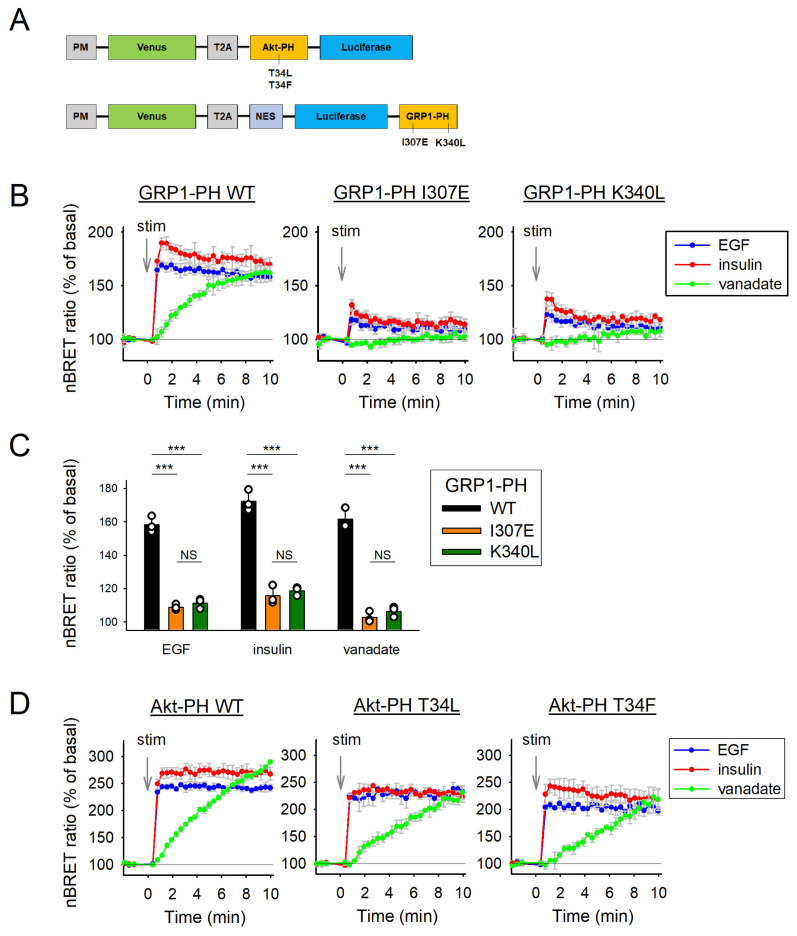
The PM PIP_3_ sensing ability of GRP1-PH- and Akt-PH-based biosensor mutants compared to the wild type (WT). (**A**) Schematic representation of the domain structures of the phosphoinositide BRET-based biosensors used in this study. ‘PM’ denotes the plasma membrane-targeting sequence of Lck, while ‘T2A’ refers to a viral peptide sequence which enables the production of two separate polypeptide chains. Mutations that disrupt protein interactions are indicated for each PH domain. (**B**) PIP_3_ level changes were measured with GRP1-PH-based BRET sensors in HEK293A cells. Cells were transiently transfected with EGFR, as well as the wild-type (WT) or mutant (I307E and K340L) sensors, as indicated. At 28 h later and after a 5 h period of serum starvation, cells were treated with 100 ng/mL EGF, 70 nM insulin or 100 nM vanadate at the zero time point (stimulation). The calculated BRET ratio values were normalized for the baselines (100%) for each curve. Data are the means ± SD, *n* = 3. (**C**) To statistically test the signals of the WT and mutant sensors with each stimulus, we calculated the means (± SD) of the last 5 measurement points of the curves from panel B. We used one-way ANOVAs with Holm–Sidak post-hoc tests separately for each treatment. *** *p* < 0.001, NS *p* > 0.05. (**D**) PIP_3_ level changes were measured with Akt-PH-based BRET sensors in HEK293A cells. Cells were transiently transfected with EGFR and either WT or mutant (T34L and T34F) sensors. Experimental conditions, treatments (EGF, insulin, and vanadate), and BRET ratio normalization were as described in panel B. Data are the means ± SD, *n* = 3.

To measure PIP_3_ levels, we utilized BRET-based biosensors by fusing the lipid-binding domains of Akt, GRP1, or their respective mutants to the BRET donor molecule luciferase. To specifically monitor PIP_3_ levels at the plasma membrane, the BRET acceptor, Venus, was anchored to the membrane using the Lck targeting sequence. Additionally, a nuclear export signal (NES) was incorporated into the GRP1-based sensors to counteract their previously observed nuclear accumulation. Both the donor and acceptor components were expressed from a single construct, separated by a T2A sequence to ensure equimolar expression following transfection. A more detailed description of this method has been published previously [22,33]. This setup enabled a comprehensive comparative analysis of biosensor behavior under various stimulations, providing insights into the role of lipid-independent interactions in biosensor dynamics. The GRP1-PH biosensor was examined alongside two mutants: K340L (lysine-to-leucine substitution at position 340) and I307E (isoleucine-to-glutamic acid substitution at position 307). Similarly, the Akt-PH sensor was analyzed with two mutants in which threonine at position 34 was replaced by leucine (T34L) or phenylalanine (T34F) (Figure 1A). These mutations in both proteins were selected in a previous study based on structural data suggesting they are located away from the lipid-binding pocket and on a putative site for protein–protein interactions (16, see also graphical abstract). All selected mutants exhibit impaired protein interactions while retaining their lipid-binding affinity [16,24].

We conducted our experiments in HEK293A cells and evaluated biosensor responses to various stimuli known to modulate PIP_3_ levels. Given the established role of receptor tyrosine kinases (RTKs) in activating PI3K and promoting PIP_3_ production, we focused on stimulation with epidermal growth factor (EGF) and insulin. To further probe PIP_3_ dynamics, we also included sodium vanadate, a phosphatase inhibitor known to elevate PIP_3_ levels. Cells were transfected with the wild type or mutant biosensors, and EGFR. After 28 h, serum-starved cells were stimulated with EGF (100 ng/mL), insulin (70 nM), or vanadate (100 nM), and BRET ratio variations were calculated.

As expected, the wild-type GRP1-PH domain displayed a significant increase in BRET ratios across all treatments, indicative of increased PM localization (Figure 1B, left graph). Conversely, the I307E and K340L mutants showed only marginal increases in BRET ratios (Figure 1B, middle and right graphs). A direct comparison of wild-type and mutant sensors following 10 min stimulation with each agent revealed a significantly reduced response in the mutants across all three treatments (Figure 1C), underscoring the importance of lipid-independent interactions in mediating the PM recruitment of the wild-type GRP1-PH domain.

Following the same experimental protocol, we then evaluated the wild-type Akt-PH domain and its two mutants, T34L and T34F. The wild-type Akt-PH domain showed a significant increase in BRET ratios across all treatments (Figure 1D, left graph), while both mutants, T34L and T34F, exhibited a comparable but slightly diminished response (Figure 1D, middle and right graphs). Taken together, these findings suggest that although the GRP1-PH domain mutants enhance lipid-binding specificity by minimizing protein-mediated interactions, their limited signal amplitude compromises their utility for dynamic lipid monitoring. In contrast, the preserved responsiveness of the Akt-PH mutants suggests that lipid-independent interactions play a lesser role in the membrane association and functional performance of Akt-based biosensors.

### 3.2. Enhancing the PIP_3_ Affinity of Lipid Selective Mutant GRP1-PH Based Biosensors

**Figure 2:**
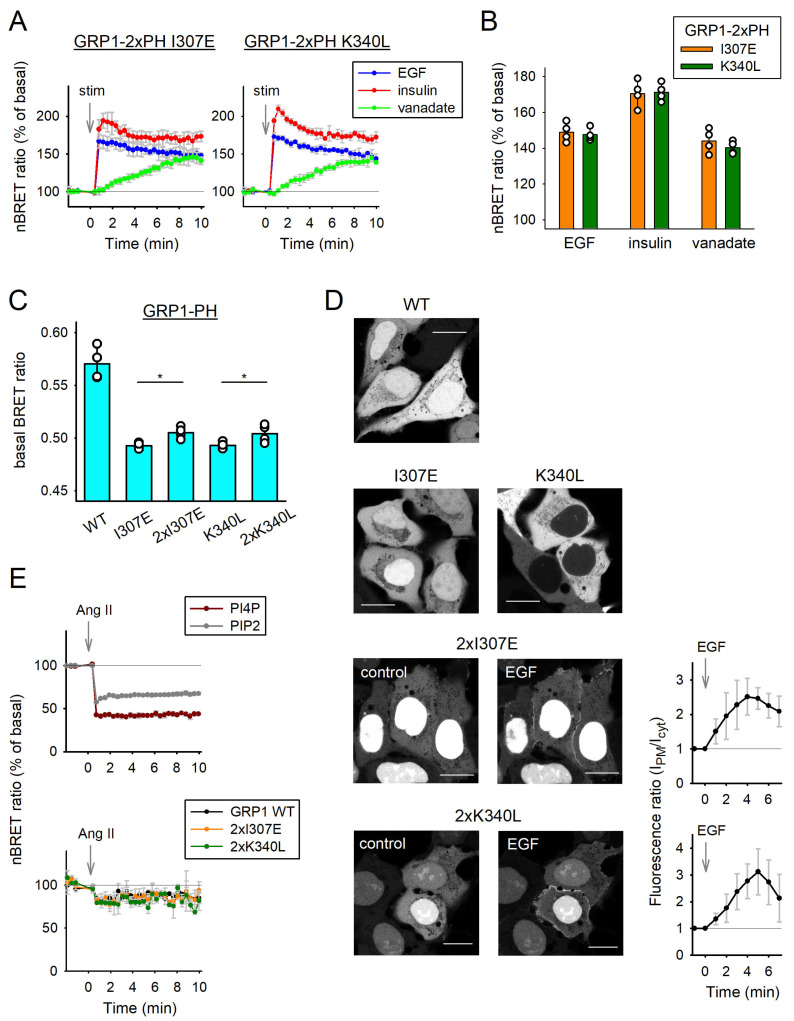
The PM PIP_3_ sensing ability of the duplicated mutant GRP1-PH based biosensors. (**A**) PIP_3_ level changes were measured using the duplicated BRET sensors in HEK293A cells. Cells were transiently transfected with EGFR and tandem mutant GRP1-PH based sensors (2xPH I307E and 2xPH K340L, as indicated). Experimental conditions, treatments (EGF, insulin, vanadate), and BRET ratio normalization were as described in Figure 1B. Data are the means ± SD, *n* = 4. (**B**) The bar graph displays the average of the last 5 measurement points of the curves from panel A, shown as means ± SD. (**C**) The bar graph shows the corresponding basal BRET ratios (before stimulation) of the indicated sensors from Figure 1B and Figure 2A. Data are the means ± SD, *n* = 4. For statistical analysis, ANOVA on Ranks with the Student–Newman–Keuls post-hoc test was used separately for each mutation site. All mutants were significantly different from the wild type. * *p* < 0.05. (**D**) Representative confocal images of serum-starved (5 h) HEK293A cells expressing the indicated lipid-binding domains tagged with Venus. Cells expressing the tandem I307E or K340L mutants were also imaged 5 min after stimulation with 100 ng/mL EGF, as indicated. Scale bar: 10 μm. After background subtraction, fluorescent intensity changes were measured. Calculation of PM and cytoplasm ratio (IPM/Icyt) was used to quantitate the extent of membrane localization (right graphs). (**E**) Changes in PI4P, PI(4,5)P_2_, and PIP_3_ levels were monitored using BRET-based biosensors in HEK293A cells. Cells were transiently transfected with a non-internalizing variant of the type 1 angiotensin receptor (AT1R Δ319) together with the GRP1-based sensors (for PIP_3_ detection) SidM-2xP4M (for PI4P) and PLCδ1-PH (for PI(4,5)P_2_), as indicated. At 28 h post-transfection and after 5 h of serum starvation, cells were stimulated with angiotensin II (100 nM). The calculated BRET ratio values were normalized to the baseline (100%) for each curve. Data represent the means ± SD, *n* = 3.

To overcome the lower affinity of the mutated GRP1-based sensors compared to the wild type, evident from their significantly diminished response to the same treatments (Figure 1C), we developed tandem versions of the previously employed mutated PH domains, an approach generally correlated with enhanced lipid binding strength. In line with the previously applied protocol, we initially transfected cells with EGFR and our novel mutated biosensors. After 28 h, serum-starved cells were treated with 100 ng/mL EGF, 70 nM insulin, or 100 nM vanadate, and BRET ratio variations were calculated. As expected, the tandem configurations of the sensors exhibited a substantially greater increase in response to each treatment (Figure 2A) compared to their single-domain counterparts (Figure 1B). To demonstrate the functionality of the tandem mutants, we averaged the BRET ratio changes observed at the end of each measurement across all stimuli (Figure 2B), which were comparable to those elicited by the wild-type sensor (Figure 1C).

To gain deeper insight into the properties of our BRET-based biosensors, we assessed their basal BRET ratios, measured before any stimulation. These initial values reflect the extent to which the lipid-binding domains are localized to the plasma membrane under resting conditions across all GRP1-PH-based sensor variants. As expected, disruption of lipid-independent interactions in the mutant sensors resulted in significantly lower basal BRET ratios compared to the wild-type GRP1-PH, indicating reduced membrane association despite intact lipid-binding capability. Notably, the tandem mutant constructs exhibited higher basal BRET ratios than their single-domain counterparts, consistent with the enhanced membrane avidity conferred by the tandem configuration (Figure 2C). Together, these results suggest that tandem GRP1-PH mutants combine enhanced membrane recruitment with independence from protein-mediated interactions, making them well-suited for reliable and specific monitoring of dynamic PIP_3_ signaling.

To further assess the utility of our newly optimized biosensors beyond BRET-based assays, we performed confocal microscopy to examine their intracellular localization and suitability for imaging-based applications. HEK293A cells were transfected with wild-type GRP1-PH, as well as the single and tandem mutant variants, each tagged with the yellow fluorescent protein Venus. The following day, serum-starved cells were imaged. All biosensors exhibited prominent cytosolic localization and, except for the single K340L mutant, the sensors were also present in the nucleus. To evaluate their responsiveness and applicability for live-cell imaging, cells expressing the tandem I307E or K340L mutants were stimulated with 100 ng/mL EGF. This induced a robust translocation of the sensors to the plasma membrane, confirming their functionality and demonstrating their suitability for confocal microscopy-based detection of PIP_3_ dynamics (Figure 2D).

Next, to assess whether the introduced mutations affected the lipid-binding specificity of our optimized GRP1-PH biosensors, particularly with regard to PI4P and PI(4,5)P_2_, we performed additional experiments targeting other plasma membrane phosphoinositides. HEK293A cells were co-transfected with wild-type or tandem GRP1-PH-based biosensors, the PI4P-specific SidM-2xP4M sensor, the PI(4,5)P_2_-specific PLCδ1-PH sensor, and a non-internalizing variant of the type 1 angiotensin receptor (AT1R Δ319) to promote pronounced changes in PM phosphoinositide levels. After 28 h, serum-starved cells were stimulated with 100 nM of angiotensin II (Ang II) and BRET ratio changes were calculated. As expected, angiotensin II treatment caused a marked reduction in PI4P and PI(4,5)P_2_ levels, as detected by the corresponding sensors (Figure 2E, upper graph). In contrast, the GRP1-based biosensors exhibited only a minor decrease in BRET signal (Figure 2E, lower graph), likely reflecting a secondary effect due to depletion of PI(4,5)P_2_, the precursor of PIP_3_. Importantly, wild-type and tandem mutant GRP1 sensors responded similarly, confirming that the introduced mutations did not alter the lipid-binding specificity of the PH domain.

### 3.3. Comparison Between Wild-Type and Mutant Sensors Under Varying Arf6 Activity Levels

Given that the mutations in our GRP1-PH sensors have been reported to disrupt interactions with activated Arf6 [16,24], we sought to assess how these sensors respond under varying levels of Arf6 activity. To this end, we repeated our BRET experiments in HEK293A cells using the wild-type (single) GRP1-PH sensor and the tandem I307E mutant, this time co-expressing either wild-type Arf6, a constitutively active GTPase-deficient mutant (Q67L), or a constitutively inactive GTP-binding–deficient mutant (T27N) [37]. To emphasize baseline differences, the resulting BRET ratios are presented without normalization.

**Figure 3:**
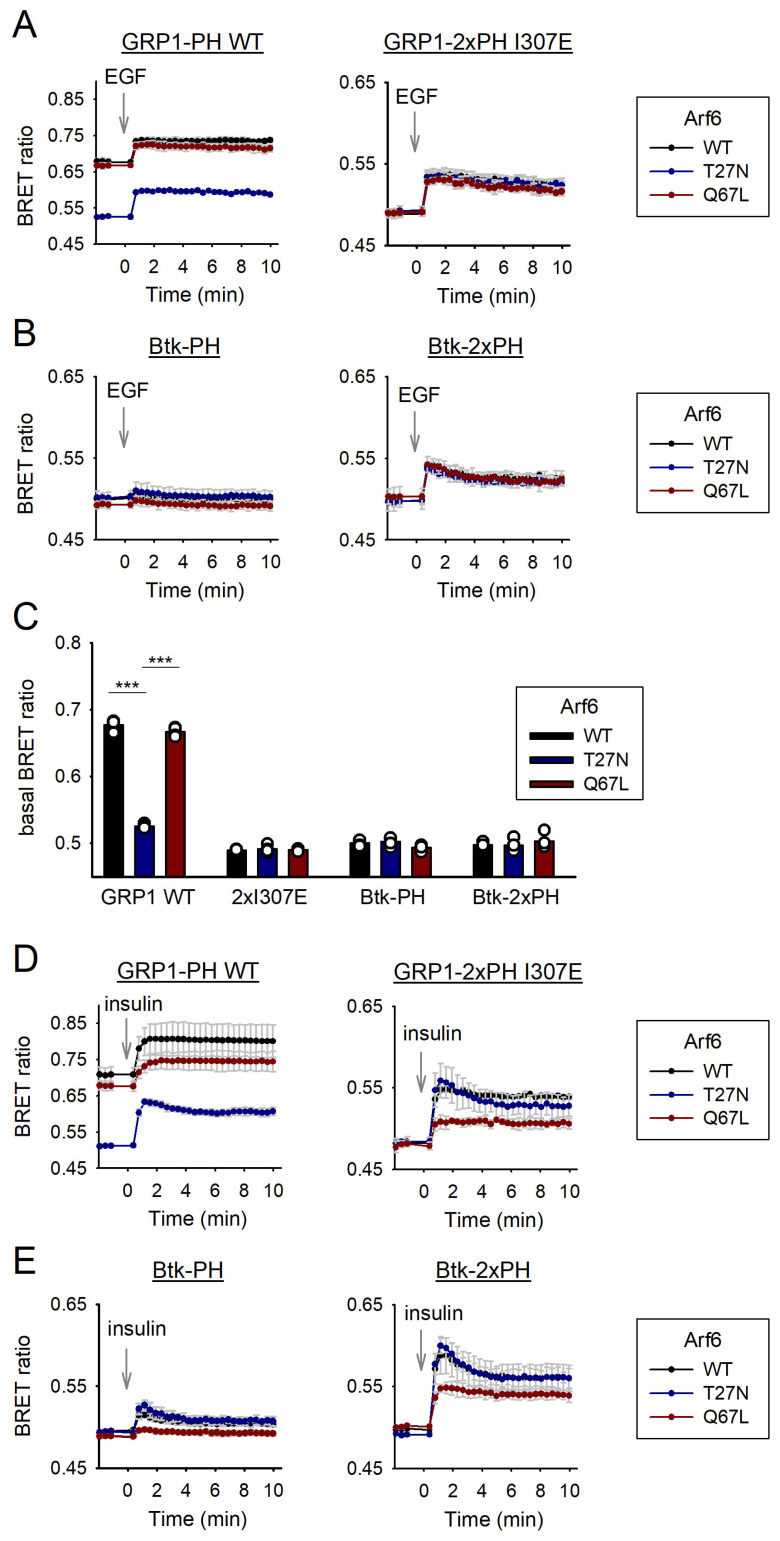
Comparison between wild-type and mutant sensors under varying Arf6 activity levels. (**A**) PIP_3_ dynamics were monitored in HEK293A cells using GRP1-PH-based BRET sensors. Cells were transiently transfected with EGFR and either wild-type or tandem I307E mutant GRP1-PH sensors, as indicated. To assess the effect of Arf6 activity, cells were also transfected with wild-type Arf6, a constitutively active GTPase-deficient mutant (Q67L), or a constitutively inactive GTP-binding-deficient mutant (T27N). At 28 h after transfection, serum starved cells (5 h) were treated with EGF (100 ng/mL), at the time point indicated by the arrows, and the BRET ratio was calculated as described in Methods. Data are the means ± SD, *n* = 4. (**B**) PIP_3_ dynamics were monitored in HEK293A cells using single and tandem Btk-PH-based BRET sensors (left and right graphs, respectively). Experimental conditions, including Arf6 constructs and stimulation, were as described in panel A. Data are the means ± SD, *n* = 4. (**C**) The bar graph shows the corresponding basal BRET ratios (before stimulation) of the indicated sensors from panels A and B. Data are the mean ± SD of 4 independent experiments. For statistical analysis, one-way ANOVA was used separately for each sensor with Tukey post-hoc test where applicable. Only significant differences are shown for each sensor. *** *p* < 0.001. (**D**) PIP_3_ dynamics were monitored in HEK293A cells using GRP1-PH-based BRET sensors. Transfection and experimental conditions were as described in panel A, except cells were stimulated with insulin (70 nM, arrow). Data are the mean ± SD, *n* = 4. (**E**) PIP_3_ dynamics were monitored in HEK293A cells using single and tandem Btk-PH-based BRET sensors (left and right graphs, respectively). Transfection and experimental conditions were as described in panel B, except cells were stimulated with insulin (70 nM, arrow). Data are the means ± SD, *n* = 4.

As shown in the left graph of Figure 3A, the wild-type GRP1-PH sensor exhibited a markedly higher basal BRET ratio when co-expressed with either wild-type Arf6 or the constitutively active Q67L mutant (black and red curves, respectively), compared to the inactive T27N variant (blue curve). In all three conditions, EGF stimulation led to a further increase in the BRET signal. In contrast, the tandem I307E mutant (right graph) displayed no appreciable differences in basal BRET ratios across the different Arf6 variants and showed a similar EGF-induced increase under all conditions.

To further contextualize our findings with the GRP1-PH-based PIP_3_ sensors, we conducted a comparative analysis using sensors based on the PH domain of Bruton’s tyrosine kinase (Btk), which is not known to be regulated by Arf6. We performed the same experimental setup as in Figure 3A, using both single and tandem versions of the Btk-PH-based sensor (Figure 3B, left and right panels, respectively). In contrast to the wild-type GRP1-PH sensor, neither sensor variant showed significant differences in basal BRET ratios across varying Arf6 activity levels, including low (T27N, blue curve), high (Q67L, red curve), or wild-type Arf6 (black curve). Consistent with our earlier observations [34], the tandem Btk-PH sensor demonstrated increased sensitivity to PIP_3_ elevation following EGF stimulation compared to the single-domain construct.

To statistically evaluate baseline BRET ratios, we averaged the pre-stimulation values for each condition and compared them separately for each sensor (Figure 3C). The wild-type GRP1 sensor showed a significantly higher basal signal when co-expressed with wild-type or constitutively active Arf6 (black and red bars, respectively) compared to the inactive T27N mutant (blue bar). In contrast, no significant differences were observed for the I307E tandem mutant or for either version of the Btk-PH-based sensor. These findings support the conclusion that plasma membrane association of the wild-type GRP1-PH domain is dependent on Arf6 activity level, and that this dependency is abolished by the I307E mutation, enhancing its specificity as a PIP_3_ sensor.

We next examined the response of the same set of PIP_3_ sensors to insulin stimulation under conditions of varying Arf6 activity (Figure 3D,E). For the wild-type GRP1-PH sensor (Figure 3D, left panel), baseline differences mirrored those observed with EGF stimulation (Figure 3A, left panel). In accordance with previously shown results, insulin elicited a stronger signal increase than EGF, although interpretation was complicated by the markedly different baseline levels across Arf6 conditions. In contrast, the tandem I307E GRP1-PH sensor (Figure 3D, right panel) showed no baseline variation across Arf6 variants, but its insulin-induced response was attenuated in the presence of constitutively active Arf6 Q67L (red curve) compared to both wild-type (black curve) and inactive T27N (blue curve) Arf6. Similar patterns were observed with the Btk-PH-based sensors (Figure 3E), where the tandem version again displayed a more pronounced response to insulin, consistent with the results seen upon EGF stimulation.

Our findings indicate that, unlike the wild-type sensor, the tandem I307E GRP1-PH-based sensor detects PIP_3_ changes independently of Arf6 activity while maintaining high sensitivity. This optimized GRP1-PH construct offers a refined biosensor system for accurate and selective monitoring of dynamic PIP_3_ signaling. Using this new sensor, alongside the Arf6-independent Btk-PH-based sensors, we also uncovered a differential insulin-induced PIP_3_ response that varies with Arf6 activity, warranting further investigation.

## 4. Discussion

In this study, we sought to refine the specificity of PIP_3_ biosensors by introducing targeted mutations into the PH domains of Akt and GRP1 that preserve lipid binding while disrupting protein-mediated interactions. Our results demonstrate that, while mutations in the Akt-PH domain had minimal impact on plasma membrane association, analogous modifications in the GRP1-PH domain significantly reduced membrane localization both before (basal) and after stimulation with EGF, insulin, and vanadate. Despite offering greater lipid specificity and diminished interference from protein–protein interactions, these single-domain GRP1 mutants exhibited limited signal amplitude, reducing their utility in tracking dynamic lipid changes. To overcome this, we developed tandem GRP1-PH mutants, which restored membrane responsiveness and achieved signal amplitudes comparable to the wild-type sensor. These optimized constructs were effective in both BRET-based assays and confocal microscopy, where EGF stimulation triggered robust translocation of the tandem K340L sensor to the plasma membrane. Moreover, unlike the wild-type sensor, the tandem GRP1-PH mutants displayed membrane recruitment independent of Arf6 activity levels, further supporting their refined specificity. Collectively, our findings establish these optimized GRP1-PH biosensors as refined tools capable of selectively and accurately reporting dynamic PIP_3_ fluctuations.

In all experiments, vanadate stimulation elicited a distinct BRET signal profile across the sensors, characterized by a continuous, progressive increase over the entire measurement period. This was in stark contrast to the rapid rise and subsequent plateau observed following EGF or insulin treatment (Figure 1B,D and Figure 2A). The unique kinetics of the vanadate response likely reflect its mode of action: rather than engaging receptor-mediated signaling cascades, vanadate exerts its effect by inhibiting phosphatases, leading to a gradual accumulation of PIP_3_ over time [38]. This distinction highlights the differential regulatory mechanisms underlying PIP_3_ dynamics depending on the nature of the upstream stimulus.

In our previous microscopy studies, we examined cells expressing fluorescent protein-tagged GRP1-PH biosensors, all of which exhibited prominent nuclear and cytosolic localization [16]. Later, we introduced the NES into the biosensors to reduce nuclear localization [34]. Surprisingly, upon introducing the NES, only the single K340L mutant showed complete exclusion from the nucleus, whereas the other sensors displayed only partial reduction in nuclear signal. Notably, the tandem sensors exhibited strong nuclear localization despite the presence of the NES (Figure 2D). This unexpected behavior suggests that the tandem configuration may impair effective nuclear export. Strong nuclear localization might be a disadvantage for these tandem mutant sensors by reducing the cytosolic fraction of the PH domain, but their enhanced responsiveness to elevations of PM PIP_3_ levels (as seen on Figure 2A after EGF and insulin stimulation) show that this can be counterbalanced by improved lipid affinity. Still, further reductions in baseline nuclear localization for these tandem mutant sensors would be desirable, e.g., by introducing tandem or multiple NES sequences.

Another possible drawback for the sensors used in this study is that they require overexpression for reliable detection, which may interfere with cellular functions by sequestering PIP_3_ or other binding partners [39]. Near endogenous levels of expression would be ideal as elegantly demonstrated in a recent study using a single-molecule detection method [40]. However, when minimizing overexpression levels and taking into account this limitation, our new sensors can still be valuable tools to monitor PIP changes and can even be adapted for single-molecule detection techniques.

Consistent with previous findings [18,24], our results indicate that, in addition to PIP_3_, active (GTP-bound) Arf6 contributes to the membrane recruitment of wild-type GRP1-PH domains, as evidenced by elevated basal signals upon overexpression of constitutively active Arf6 (Q67L) (Figure 3A,C). Notably, overexpression of wild-type Arf6 produced similarly high baseline signals, suggesting that a substantial proportion of Arf6 is in its active state under these conditions. In contrast, the tandem I307E mutant sensor showed no sensitivity to changes in Arf6 activity levels and displayed reduced membrane association compared to the wild-type sensor even in the presence of inactive Arf6 (T27N), raising the possibility that high levels of inactive Arf6 may still contribute to GRP1-PH membrane localization. However, the physiological relevance of this observation remains unclear and warrants further investigation.

Another notable observation from these experiments was the differential effect of Arf6 activity on insulin-induced PIP_3_ signal elevation, which was consistent across all PIP_3_ biosensors tested. Interestingly, overexpression of wild-type or inactive Arf6 resulted in comparable increases in PIP_3_ levels following insulin stimulation, whereas constitutively active Arf6 attenuated this response. This finding is particularly valuable, as it suggests that these PIP_3_ biosensors can also be used to indirectly assess Arf6 activity states in the context of insulin signaling. While both GRP1 and Arf6 have been previously implicated in insulin signaling downstream of PI3K and Akt [41], the mechanisms by which Arf6 modulates insulin-induced PIP_3_ production remain unclear and warrant further investigation beyond the scope of this study.

## 5. Conclusions

In this study, we developed refined GRP1-PH-based biosensors to improve the specificity and performance of PIP_3_ detection at the plasma membrane. By introducing targeted mutations that disrupt protein–protein interactions while preserving lipid binding, we generated single-domain variants with enhanced selectivity but reduced signal amplitude. To overcome this limitation, we engineered tandem versions of these mutants, which restored membrane responsiveness to levels comparable with the wild-type sensor. Despite their increased nuclear localization, these tandem constructs demonstrated robust performance in both live-cell imaging and BRET-based assays, offering a reliable tool for dynamic and selective monitoring of PIP_3_ signaling with minimal interference from non-lipid interactions.

Our experiments also evaluated the contributions of Arf6 activity to GRP1-PH membrane localization and PIP_3_ signaling. The wild-type GRP1-PH sensor displayed elevated basal membrane association when co-expressed with wild-type or constitutively active Arf6, supporting the role of the active Arf6 in facilitating membrane recruitment. In contrast, the tandem I307E mutant showed no sensitivity to Arf6 activity, confirming its specificity for PIP_3_ and independence from protein-mediated interactions. Taken together, our results demonstrate that the optimized GRP1-PH constructs serve as reliable and specific tools for monitoring dynamic changes in PIP_3_ levels, with reduced susceptibility to interference from protein-mediated interactions.

## Data Availability

The datasets generated and/or analyzed during the current study are available from the corresponding author upon reasonable request.

## Abbreviations

The following abbreviations are used in this manuscript:

Akt	Protein kinase B
Ang II	Angiotensin II
Arf6	ADP ribosylation factor 6
AT1R	Type I angiotensin receptor
BRET	Bioluminescence resonance energy transfer
Btk	Bruton’s tyrosine kinase
DMEM	Dulbecco’s modified Eagle medium
EGF	Epidermal growth factor
EGFR	Epidermal growth factor receptor
GAP	GTPase-activating protein
GEF	Guanine nucleotide exchange factor
GRP1	General receptor for phosphoinositides 1
NES	Nuclear export signal
PH-domain	Pleckstrin homology-domain
PI	Phosphoinositides
PI3K	Phosphoinositide 3-kinase
PIP	Phosphatidylinositol phosphate
PI4P	Phosphatidylinositol 4-phosphate
PI(4,5)P_2_	Phosphatidylinositol 4,5-bisphosphate
PIP_3_	Phosphatidylinositol 3,4,5-trisphosphate
PM	Plasma membrane
RTK	Receptor tyrosine kinase
WT	Wild type
